# Prevalence and Risk Factors of Subclinical and Overt Hypothyroidism in Saudi Arabia: A Systematic Review

**DOI:** 10.7759/cureus.86336

**Published:** 2025-06-19

**Authors:** Ali Hadi M Alhajri, Anoud Mubarak Saad Saleh Al Saad, Hoda Idrees, Osama Mohamed Hamednalla Mohamed, Mona Mohammednoor, Ibrahim Obied Ibrahim Ahmed, Fatima Suliman Dawod Faky

**Affiliations:** 1 Endocrinology, Najran Armed Forces Hospital, Ministry of Defense Health Services, Najran, SAU; 2 General Practice, Khamis Mushait General Hospital, Khamis Mushait, SAU; 3 General Practice, West Midlands Deanery, Birmingham, GBR; 4 Internal Medicine, Ibri Regional Hospital, Ibri, OMN; 5 Diabetes and Endocrinology, Queen Elizabeth Hospital Birmingham, Birmingham, GBR; 6 Internal Medicine, Prince Miteb bin Abdulaziz Hospital, Saka, SAU; 7 Clinical Pathology, Hafar Al-Batin Central Hospital, Hafar Al-Batin, SAU

**Keywords:** hypothyroidism, prevalence, risk factors, saudi arabia, subclinical hypothyroidism, thyroid disorders

## Abstract

Hypothyroidism, whether subclinical (SCH) or overt (OH), stands among the most common yet often overlooked endocrine disorders, with its prevalence varying widely across different populations. In Saudi Arabia, factors such as metabolic comorbidities, genetic predisposition, and regional disparities contribute to its epidemiology. This systematic review aimed to summarize the existing literature on the prevalence and risk factors of SCH and OH in Saudi Arabia to lead clinical practice and public health strategies. A comprehensive literature search was conducted to identify relevant studies published in the past decade. Studies meeting predefined inclusion criteria were selected, and data on study characteristics, prevalence, risk factors, and diagnostic methods were extracted. Methodological quality was assessed using a standardized appraisal tool. Due to heterogeneity, a narrative synthesis was performed. Reported prevalence rates varied across populations, with higher rates observed in certain groups such as pregnant women and individuals with specific comorbidities. Female gender, advanced age, family history, and chronic conditions were associated risk factors. Broader screening approaches in pregnancy appeared to identify more SCH cases than selective strategies. Regional differences in prevalence within Saudi Arabia were also noted. Most included studies demonstrated low to moderate risk of bias. The burden of hypothyroidism in Saudi Arabia varies by population and region, with distinct risk factors underscoring the need for tailored screening and management. Expanded screening in high-risk groups and further research on causal pathways are recommended to address this growing health concern.

## Introduction and background

Thyroid disorders represent a significant global health burden, with hypothyroidism being one of the most prevalent endocrine conditions worldwide [1]. The spectrum of hypothyroidism ranges from overt disease, characterized by elevated thyroid-stimulating hormone (TSH) and reduced free thyroxine (FT4) levels, to subclinical hypothyroidism (SCH), defined by elevated TSH with normal FT4 concentrations [2]. While overt hypothyroidism presents with clear clinical manifestations, SCH often remains asymptomatic yet may progress to overt disease and has been associated with adverse cardiovascular and metabolic outcomes, particularly in vulnerable populations such as pregnant women and the elderly [3].

In Saudi Arabia, the epidemiology of thyroid disorders presents unique characteristics that warrant careful examination. The Kingdom's rapid urbanization, dietary transitions, and high prevalence of metabolic disorders such as obesity and diabetes mellitus create a distinct context for thyroid dysfunction [4]. Furthermore, regional variations in iodine status and genetic predisposition may contribute to geographical differences in disease prevalence [5]. Recent studies from various regions of Saudi Arabia have reported widely varying prevalence rates of SCH with even higher rates observed in specific subgroups such as pregnant women and obese individuals [6]. These variations highlight the need for a comprehensive summary of existing literature to clarify the true burden of disease.

The clinical significance of understanding hypothyroidism patterns in Saudi Arabia extends beyond epidemiological interest. Accurate prevalence data and risk factor identification are crucial for developing appropriate screening strategies, particularly for high-risk groups [7]. Current clinical practices regarding thyroid function testing and management of SCH vary considerably across healthcare institutions in the Kingdom, reflecting ongoing debates in the international medical community about optimal diagnostic thresholds and treatment indications [8]. This variability in clinical approach underscores the importance of establishing an evidence base tailored to the Saudi population's specific characteristics and healthcare needs.

Despite numerous individual studies investigating hypothyroidism prevalence in different Saudi populations, there remains a lack of comprehensive systematic review that summarizes the existing literature. Previous reviews have either focused narrowly on specific subgroups (such as pregnant women) or have not systematically evaluated the quality of included studies [4,9,10]. This systematic review therefore aims to provide a rigorous and comprehensive summary of existing evidence on the prevalence and risk factors of both subclinical and overt hypothyroidism across various populations in Saudi Arabia. By employing standardized quality assessment tools and a transparent methodology, we seek to establish reliable estimates of disease burden while identifying key demographic and clinical factors associated with thyroid dysfunction in the Saudi population. The findings will contribute to ongoing discussions about optimal screening approaches and may inform the development of national guidelines for thyroid disorder management in Saudi Arabia, ultimately aiming to improve endocrine health outcomes across the Kingdom.

## Review

Methodology

Design and Aim of Study

This systematic review was conducted in accordance with the Preferred Reporting Items for Systematic Reviews and Meta-Analyses (PRISMA) 2020 guidelines [11] to ensure transparency, reproducibility, and methodological rigor. The review aimed to synthesize evidence on the prevalence and risk factors of SCH and overt hypothyroidism (OH) in Saudi Arabia.

Eligibility Criteria

Studies were selected based on predefined inclusion and exclusion criteria to maintain consistency and relevance. The criteria were established before the literature search to minimize selection bias. The detailed inclusion and exclusion criteria are provided in Table 1.

**Table 1:** Inclusion and Exclusion Criteria SCH: subclinical hypothyroidism, OH: overt hypothyroidism.

Category	Inclusion Criteria	Exclusion Criteria
Population	Human populations in Saudi Arabia	Non-Saudi populations or animal studies
Study design	Observational studies (cross-sectional, cohort, case-control)	Reviews, case reports, editorials, conference abstracts
Outcomes	Reports on prevalence or risk factors of SCH/OH	No data on SCH/OH prevalence or risk factors
Language	English or Arabic	Other languages
Publication	Studies published from 2015 to 2025	Studies before 2015

Information Sources and Search Strategy

A comprehensive literature search was conducted across five electronic databases: PubMed, Scopus, Web of Science, ScienceDirect, and Cochrane Library. The search strategy was designed to capture all relevant studies using a combination of keywords and Medical Subject Headings (MeSH) terms related to hypothyroidism, prevalence, risk factors, and Saudi Arabia (Table 2). The search was limited to human studies and studies published from 2015 to 2025 in order to include the recent literature on the subject.

**Table 2:** Search Strings for Each Database

Database	Search String
PubMed	("Hypothyroidism"[Mesh] OR "Subclinical Hypothyroidism"[Mesh]) AND ("Saudi Arabia"[Mesh] OR "KSA") AND ("Prevalence"[Mesh] OR "Risk Factors"[Mesh])
Scopus	TITLE-ABS-KEY (hypothyroidism OR "subclinical hypothyroidism") AND TITLE-ABS-KEY ("Saudi Arabia" OR "KSA") AND TITLE-ABS-KEY (prevalence OR "risk factors")
Web of Science	TS=("hypothyroidism" OR "subclinical hypothyroidism") AND TS=("Saudi Arabia" OR "KSA") AND TS=("prevalence" OR "risk factors")
ScienceDirect	("hypothyroidism" OR "subclinical hypothyroidism") AND ("Saudi Arabia" OR "KSA") AND ("prevalence" OR "risk factors")
Cochrane	(hypothyroidism OR "subclinical hypothyroidism") AND ("Saudi Arabia" OR "KSA") AND (prevalence OR "risk factors")

The search was supplemented by manually reviewing the reference lists of included studies to identify additional relevant articles that might have been missed in the database searches.

Study Selection Process

The study selection process followed the PRISMA 2020 flow diagram to ensure transparency and reproducibility. After removing duplicates, two independent reviewers (AHMA and FSDF), from the list of authors, screened the titles and abstracts of all retrieved records to identify potentially eligible studies. Full-text articles of the selected abstracts were then assessed for eligibility based on the predefined criteria. Any discrepancies between reviewers were resolved through discussion or consultation with a third reviewer (OMHM), who served as a tiebreaker. The final list of included studies was agreed upon by consensus.

Data Extraction and Management

Data from eligible studies were systematically extracted using a standardized form to ensure consistency and accuracy. The extracted information included study characteristics (author, year, design, location, sample size), population details (age, gender, subgroups), outcome measures (prevalence of SCH/OH, diagnostic criteria, risk factors), and key findings (significant associations, statistical methods, limitations). Two independent reviewers (AHMA and FSDF) performed the extraction to minimize errors, with any discrepancies resolved through discussion. All extracted data were cross-checked for accuracy prior to summarization by the third reviewer (OMHM).

Risk of Bias Assessment

The methodological quality of included studies was rigorously evaluated using the Newcastle-Ottawa Scale (NOS) [12] adapted for cross-sectional studies. This tool assessed three critical domains: selection (evaluating sample representativeness, size, non-response rate, and exposure ascertainment), comparability (examining adjustment for key confounders like age, sex, and comorbidities), and outcome/exposure (analyzing assessment methods and statistical appropriateness). Each study received a score out of 10, with higher scores (≥8) indicating low risk of bias, moderate scores (6-7) suggesting moderate risk, and lower scores (≤5) reflecting high risk. Two independent reviewers (AHMA and FSDF) conducted the assessments to ensure objectivity, with any discrepancies resolved through consensus discussion to maintain the review's integrity. This systematic approach provided a robust framework for evaluating study quality while minimizing potential bias in the review process.

Data Synthesis

Due to substantial heterogeneity across studies in design, population characteristics, diagnostic criteria, and outcome reporting, we conducted a qualitative synthesis rather than meta-analysis. This methodological decision prevented potentially misleading pooled estimates while allowing for meaningful comparison of patterns and discrepancies through narrative synthesis. The approach adheres to PRISMA 2020 guidelines for handling heterogeneous evidence, ensuring a rigorous yet flexible interpretation of findings within their proper clinical and methodological contexts.

Results

Study Selection

**Figure 1:**
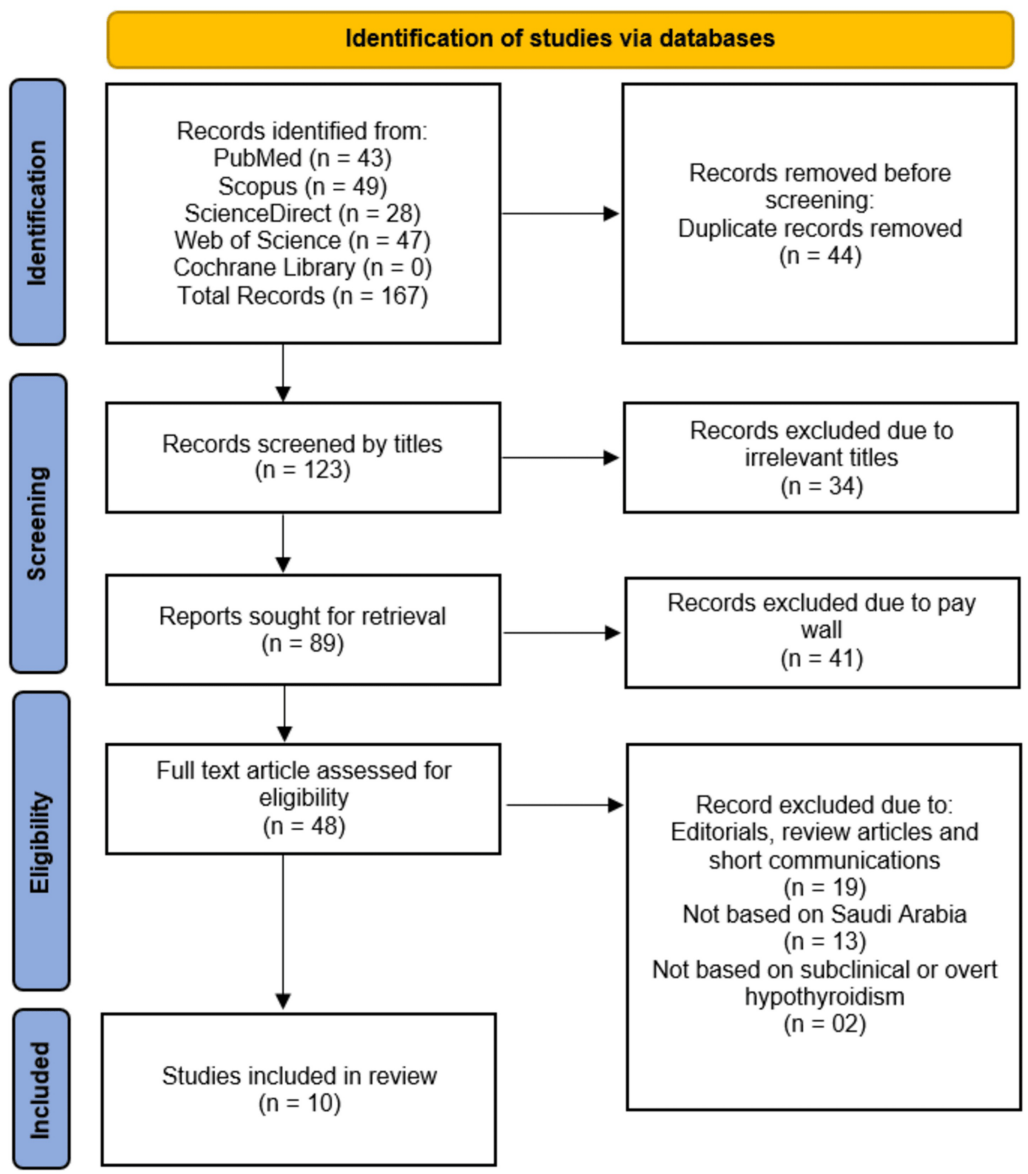
PRISMA Flow Diagram Showing Studies Selection Process PRISMA: Preferred Reporting Items for Systematic Reviews and Meta-Analyses.

The initial search across five databases (PubMed, Scopus, ScienceDirect, Web of Science, and Cochrane Library) yielded 167 records, from which 44 duplicates were removed. After screening the remaining 123 records by title, 34 were excluded as irrelevant. Of the 89 records sought for retrieval, 41 were excluded due to paywall restrictions, 19 were editorials/reviews/short communications, 13 were not based on Saudi Arabia, and two did not focus on subclinical or overt hypothyroidism. Ultimately, 48 full-text articles were assessed for eligibility, with 10 studies meeting the inclusion criteria for the review (Figure 1).

Prevalence of Subclinical and Overt Hypothyroidism 

We included eight studies [9,10,13-18] from different location in Saudi Arabia. The included studies reported varying prevalence rates of SCH and OH across different populations in Saudi Arabia. Among pregnant women, the prevalence of SCH ranged from 10.4% to 15.9% [9,13,15], with one study noting a significantly higher likelihood of SCH in women screened randomly compared to those referred by physicians (OR: 3.1; 95% CI: 1.182-8.704; p=0.022) [13]. In the general adult population, SCH prevalence was reported as 10.3% in primary healthcare visitors [14] and 15.9% in individuals undergoing thyroid function tests [9]. Notably, OH was less common, with rates of 0% in primary healthcare settings [14] and 1.3% among pregnant women [15]. However, studies focusing on specific populations, such as patients with obesity hypoventilation syndrome (OHS), reported higher OH prevalence (18.8%) [18]. The overall hypothyroidism prevalence (combining SCH and OH) ranged from 18.7% to 25.5% in general population studies [10,16,17], with females disproportionately affected (Table 3).

**Table 3:** Characteristics of Included Studies SCH: subclinical hypothyroidism, OH: overt hypothyroidism, PHC: primary health care, BMI: body mass index, TSH: thyroid-stimulating hormone, FT4: free thyroxine, FT3: free triiodothyronine, USPSTF: United States Preventive Services Task Force, ATA: American Thyroid Association, OHS: obesity hypoventilation syndrome, OSA: obstructive sleep apnea, M/F: male/female.

Study	Study Location	Study Design	Sample Size	Population Description	Age Range/Mean Age	Gender (M/F)	Prevalence of SCH (%)	Prevalence of OH (%)	Risk Factors Assessed	Diagnostic Criteria Used	Key Findings
Al Shanqeeti et al., [13] (2018)	Riyadh, Saudi Arabia (King Abdulaziz Medical City and Khashmulaan Clinic, National Guard Health Affairs)	Cross-sectional	384	Pregnant women attending antenatal clinics	Not explicitly stated; includes ≥40 years category	0/384 (F)	13% (50/384)	Not reported	Age ≥40 years (not significant); Screening method (random vs. physician referral)	Not reported	SCH prevalence was higher in women randomly screened vs. physician referred; women randomly screened were 3x more likely to have SCH. Highlights need for broader screening.
Al Eidan et al., [14] (2018)	Riyadh, Saudi Arabia	Cross-sectional	394 (340 gave blood samples)	Adult visitors to nine PHC satellite clinics in military housing	Not specified; elderly defined as ≥60 years	Not specified	10.3%	0%	Age, gender, family history, BMI, hyperlipidemia, blood pressure	Elevated TSH with normal free T4 (SCH); overt hypothyroidism not detected	SCH prevalence was 10.3%; higher TSH levels in ≥60 years and those with family history; no OH detected; upward TSH trend with hyperlipidemia and BP.
Fureeh et al., [9] (2019)	Al-Baha, Saudi Arabia	Retrospective cross-sectional study	567 (from 1,000 reviewed)	Patients visiting central laboratory over eight years with thyroid function test results	Not specified	Not fully reported (higher prevalence in females)	15.9%	Not assessed (no overt hypothyroidism reported)	Gender (female), Age (elderly)	Based on laboratory standard values: TSH level with normal free T4 and free T3 for SCH	Subclinical hypothyroidism prevalence was 15.9%, higher in females (13.2%). Subclinical thyroid disease is common and more prevalent in elderly females.
Zahrani et al., [15] (2022)	Saudi Arabia (four antenatal clinics)	Multicenter, cross-sectional	386	Pregnant women attending routine antenatal visits	Not specified	Female only	10.4%	1.3%	Risk stratification based on USPSTF and ATA criteria	TSH and T4 levels (thyroid function tests)	Prevalence of dysthyroidism was 11.7%; targeted screening based on USPSTF and ATA criteria showed poor performance; universal screening suggested.
Alqahtiani et al., [10] (2020)	Saudi Arabia (various regions)	Cross-sectional	2,417	General population from different areas of Saudi Arabia	Not specified	Majority female (86.3% of hypothyroid cases)	Not specified	18.7% (total hypothyroidism; SCH/OH not separated)	Age, sex, family history, obesity, autoimmune diseases, diabetes mellitus	Not explicitly stated	Hypothyroidism prevalence was 18.7%; 15.3% of hypothyroid cases had diabetes. Significant associations found with age, sex, family history, obesity, autoimmune diseases. Most cases were female.
Mahfouz, [16] (2021)	Saudi Arabia	Cross-sectional	981	General Saudi population	Not reported	Not reported	Not separately reported	20.6% (overall hypothyroidism)	Family history of hypothyroidism, ovarian cysts (women), diabetes mellitus, obesity, smoking, dietary habits (coffee, sugary foods, soft drinks), exophthalmos, swollen neck	Self-reported diagnosis via online questionnaire	High prevalence (20.6%) of hypothyroidism found; ovarian cysts (in women) and family history of hypothyroidism identified as significant risk factors via logistic regression analysis.
Alrowaili et al., [17] (2018)	Arar city, Northern Saudi Arabia	Cross-sectional	454	General population of Arar city	21-60 years (80% of cases)	57.7% female of hypothyroid cases	Not specified	25.5% overall hypothyroidism	Family history (40%), sex (female predominance), treatment response	Self-reported questionnaire (no clinical/lab diagnosis)	High hypothyroidism prevalence (25.5%), females more affected, 40% family history, 64.7% on treatment with low response.
BaHammam et al., [18] (2020)	Saudi Arabia	Prospective observational	308 (OHS group)	Patients with obesity hypoventilation syndrome (OHS)	Mean age 55.1 ± 13.8 years	Matched groups, exact M/F not specified	6.2%	18.8%	Female sex (as predictor of OH)	Elevated TSH + normal FT4 for SCH; elevated TSH + low FT4 for OH	Clinical hypothyroidism prevalent in OHS patients; female sex was the only significant predictor of OH; SCH more common in OHS than OSA patients.

Associated Risk Factors

Several risk factors were consistently associated with SCH and OH. Female gender emerged as a significant predictor across multiple studies [9,10,18], with logistic regression analysis in one study identifying female sex as the sole significant predictor of OH (OR: 2.801; 95% CI: 1.386-5.662; p=0.004) [18]. Advanced age, particularly ≥60 years, was linked to higher SCH prevalence (p=0.047) [14]. Family history of thyroid disease was another prominent risk factor, with 40% of hypothyroid cases reporting a positive family history in one study [17]. Additionally, comorbidities such as obesity [10], autoimmune diseases [10], and diabetes mellitus (p=0.003) [10] were significantly associated with hypothyroidism. In women, ovarian cysts and dietary habits (e.g., high sugar intake) were also identified as risk factors [16].

Diagnostic and Screening Practices

Diagnostic criteria and screening methods varied among studies. Most studies defined SCH based on elevated thyroid-stimulating hormone (TSH) levels with normal free thyroxine (T4) [9,14,18]. However, some studies relied on self-reported diagnoses or questionnaires, which may introduce bias [16,17]. Targeted screening based on U.S. Preventive Services Task Force (USPSTF) and American Thyroid Association (ATA) criteria showed poor performance in identifying high-risk pregnant women, leading to calls for universal screening [15]. The higher prevalence of SCH in randomly screened pregnant women further supports this recommendation [13] (Table 4).

**Table 4:** Prevalence and Associated Risk Factors for Subclinical and Overt Hypothyroidism SCH: subclinical hypothyroidism, OH: overt hypothyroidism, USPSTF: United States Preventive Services Task Force, ATA: American Thyroid Association, OHS: obesity hypoventilation syndrome, OSA: obstructive sleep apnea.

Author (s) (Year)	SCH Prevalence (%)	OH Prevalence (%)	Subgroup Analysis	Significant Risk Factors (Adjusted)	Statistical Methods Used
Al Shanqeeti et al., [13] (2018)	13% (50/384)	Not reported	Pregnant women screened randomly vs. physician referral	Random screening (OR: 3.1; 95% CI: 1.182-8.704; p=0.022)	Odds ratio (OR), 95% confidence interval (CI), p-value
Al Eidan et al., [14] (2018)	10.3%	0%	Age ≥60 years, family history of thyroid disease, comorbidities	Age ≥60 years (p = 0.047), family history of thyroid disease (p=0.047)	Chi-square, t-test, ANOVA, linear regression
Fureeh et al., [9] (2019)	15.9	Not reported	Gender (female vs. male)	Female gender	Not specified
Zahrani et al., [15] (2022)	10.4%	1.3%	Comparison between low-risk vs. high-risk groups based on USPSTF and ATA criteria	Not reported in adjusted form	Sensitivity, specificity, accuracy calculations for screening performance
Alqahtiani et al., [10] (2020)	Not specified	18.7% (total hypothyroidism, likely includes OH and possibly SCH)	Age, sex, diabetes type, family history, obesity, autoimmune diseases	Age, sex, family history of hypothyroidism, obesity, autoimmune diseases, diabetes (p=0.003)	Chi-square test (implied by p-values), possibly multivariate analysis (not clearly stated)
Mahfouz [16] (2021)	Not specified	Not specified	Women with DM, exophthalmos, swollen neck, obesity, smokers, family history, ovarian cyst, elevated lactation, dietary habits (coffee, sugary foods, soft drinks)	Ovarian cysts (women), family history of hypothyroidism	Binary logistic regression
Alrowaili et al., [17] (2018)	Not reported	25.5%	Females (57.7%), age 21-60 years (80%), family history (40%)	Family history and female sex (no adjustment reported)	Not specified; descriptive cross-sectional analysis
BaHammam et al., [18] (2020)	6.2	18.8	Comparison between OHS and OSA patients; subclinical hypothyroidism more common in OHS (6.2% vs. 2.9%, p=0.03)	Female sex (OR: 2.801, 95% CI: 1.386-5.662, p=0.004) for clinical hypothyroidism	Logistic regression model; comparison tests (p-values)

Subgroup Variations

Subgroup analyses revealed notable variations in prevalence and risk factors. For instance, SCH was more common in OHS patients (6.2%) compared to those with obstructive sleep apnea (2.9%; p=0.03) [18]. Pregnant women screened universally had higher SCH detection rates than those screened selectively [13]. Additionally, studies in northern Saudi Arabia (e.g., Arar City) reported higher hypothyroidism prevalence (25.5%) compared to national averages [17], suggesting regional disparities.

Risk of Bias Findings

Six studies demonstrated low risk of bias, with Al Eidan et al. [14], Zahrani et al. [15], and BaHammam et al. [18] achieving the highest scores (9/10) due to their robust study designs, clear diagnostic criteria, and appropriate control for confounding factors. Al Shanqeeti et al. [13] and Alqahtiani et al. [10] scored slightly lower (8/10) primarily due to minor limitations in outcome assessment. Fureeh et al. [9] (7/10) and Alrowaili et al. [17] (6/10) were rated as moderate risk due to issues with sample representativeness and limited adjustment for confounders. Mahfouz [16] (5/10) showed moderate risk of bias, mainly because of its reliance on self-reported data without clinical validation and inadequate sample size justification (Table 5).

**Table 5:** Risk of Bias Results Using NOS Tool NOS: Newcastle-Ottawa Scale.

Study	Selection (Max 5)	Comparability (Max 2)	Outcome/Exposure (Max 3)	Total Score (Max 10)	Risk of Bias
Al Shanqeeti et al., [13] (2018)	★★★★☆ (4)	★★ (2)	★★☆ (2)	8/10	Low
Al Eidan et al., [14] (2018)	★★★★☆ (4)	★★ (2)	★★★ (3)	9/10	Low
Fureeh et al., [9] (2019)	★★★☆☆ (3)	★☆ (1)	★★★ (3)	7/10	Low
Zahrani et al., [15] (2022)	★★★★☆ (4)	★★ (2)	★★★ (3)	9/10	Low
Alqahtiani et al., [10] (2020)	★★★★☆ (4)	★★ (2)	★★☆ (2)	8/10	Low
Mahfouz [16] (2021)	★★☆☆☆ (2)	★☆ (1)	★★☆ (2)	5/10	Moderate
Alrowaili et al., [17] (2018)	★★★☆☆ (3)	★☆ (1)	★★☆ (2)	6/10	Moderate
BaHammam et al., [18] (2020)	★★★★☆ (4)	★★ (2)	★★★ (3)	9/10	Low

Discussion

The findings of this systematic review provide a comprehensive overview of the prevalence and risk factors associated with SCH and OH in Saudi Arabia, synthesizing data from eight studies conducted across diverse populations. The results reveal significant variability in the burden of thyroid dysfunction, with SCH prevalence ranging from 6.2% to 15.9% and OH from 0% to 25.5%, depending on the population studied. Among pregnant women, SCH prevalence was notably high (10.4%-15.9%) [9,13,15], aligning with global trends that identify pregnancy as a vulnerable period for thyroid dysfunction due to hormonal fluctuations and increased metabolic demands [19]. The observation that universal screening detected higher SCH rates compared to targeted approaches (OR: 3.1; 95% CI: 1.182-8.704) [13] underscores the limitations of risk-based screening strategies, a finding consistent with international studies advocating for universal thyroid screening in pregnancy to mitigate adverse maternal and fetal outcomes [20].

In the general adult population, the prevalence of SCH (10.3%-15.9%) [9,14] was comparable to rates reported in other Middle Eastern countries, such as Iran (12.5%) and Turkey (11.7%) [21,22], but higher than in Western populations (4%-8%) [23]. This disparity may reflect regional differences in iodine status, genetic predisposition, or diagnostic thresholds. Notably, OH was rare in primary care settings (0%) [14] but elevated in specialized cohorts, such as patients with obesity hypoventilation syndrome (18.8%) [18]. The latter finding aligns with evidence linking severe obesity to hypothalamic-pituitary-thyroid axis dysregulation [24], suggesting that metabolic comorbidities may exacerbate thyroid dysfunction. The overall hypothyroidism prevalence (18.7%-25.5%) [10,16,17] exceeded global estimates (5%-15%) [25], potentially due to high rates of risk factors like obesity and diabetes in Saudi Arabia [26].

Female gender emerged as a consistent predictor of hypothyroidism across studies [9,10,18], with BaHammam et al. [18] reporting a 2.8-fold higher odds of OH in women (95% CI: 1.386-5.662). This aligns with well-established sex disparities in autoimmune thyroid diseases, particularly Hashimoto’s thyroiditis, which disproportionately affect women due to estrogen-mediated immune modulation [27]. Advanced age (≥60 years) was another robust risk factor [14], consistent with the natural decline in thyroid function with aging and the accumulation of autoimmune insults [28]. Family history of thyroid disorders, reported in 40% of cases in Arar City [17], further highlights the role of genetic predisposition, as evidenced by twin studies estimating a 55%-65% heritability for hypothyroidism [29].

Comorbidities such as obesity, diabetes, and autoimmune diseases were significantly associated with hypothyroidism [10], mirroring global data on metabolic and immune-mediated pathways in thyroid dysfunction [30]. The link between ovarian cysts and hypothyroidism in women [16] may reflect shared endocrine disruptions, particularly polycystic ovary syndrome (PCOS), which is often comorbid with autoimmune thyroiditis [31]. However, the reliance on self-reported data in Mahfouz [16] limits causal inference, as recall bias and misclassification could inflate these associations. Dietary factors, including high sugar intake, were also implicated [16], although mechanistic evidence remains sparse. These findings collectively underscore the multifactorial etiology of hypothyroidism, necessitating integrated clinical and public health strategies.

Diagnostic and screening practices varied widely, with most studies defining SCH biochemically (elevated TSH with normal T4) [9,14,18], while others used self-reports [16,17]. The latter approach, though pragmatic for large surveys, risks underestimating true prevalence due to underdiagnosis or overestimating it due to misclassification. The poor performance of USPSTF/ATA criteria in identifying high-risk pregnant women [15] challenges current screening guidelines, echoing debates in the U.S. and Europe about the cost-effectiveness of universal vs. targeted screening [32]. The higher SCH yield with universal screening [13] supports calls for standardized, population-wide approaches, particularly in high-prevalence regions like Saudi Arabia.

Geographic disparities were evident, with northern Saudi Arabia (Arar City) reporting a 25.5% hypothyroidism prevalence [17], nearly double the national average. This could reflect regional iodine deficiency, genetic factors, or environmental triggers (e.g., selenium deficiency), although further research is needed to clarify these drivers [33]. Similarly, the higher SCH prevalence in OHS vs. OSA patients (6.2% vs. 2.9%; p=0.03) [18] suggests that severe obesity-related hypoxia may uniquely disrupt thyroid function, a hypothesis warranting mechanistic studies.

Limitations

This review has several limitations. First, the included studies exhibited methodological heterogeneity in design, sampling, and diagnostic criteria, complicating direct comparisons. For instance, Mahfouz [16] and Alrowaili et al. [17] relied on self-reports, introducing potential misclassification bias. Second, most studies were cross-sectional, precluding causal inferences. Third, regional variability in iodine status and healthcare access, unmeasured in many studies, may confound prevalence estimates. Finally, publication bias could skew the literature toward higher-prevalence estimates, as negative findings are often underreported.

## Conclusions

The prevalence of SCH and OH varies widely by population, with pregnancy and severe obesity emerging as high-risk conditions. Female sex, family history, and comorbidities like diabetes and autoimmune diseases are consistent risk factors, underscoring the need for targeted screening and multidisciplinary management. The poor performance of risk-based screening in pregnancy and the regional disparities in prevalence call for national guidelines advocating universal screening in high-risk groups and regions. Future research should prioritize longitudinal designs to elucidate causal pathways and evaluate the impact of interventions, such as iodine fortification or lifestyle modifications, on thyroid health in Saudi Arabia. By addressing these gaps, policymakers and clinicians can mitigate the growing burden of thyroid dysfunction in the region.
